# Conformal High-K Dielectric Coating of Suspended Single-Walled Carbon Nanotubes by Atomic Layer Deposition

**DOI:** 10.3390/nano9081085

**Published:** 2019-07-28

**Authors:** Aidar Kemelbay, Alexander Tikhonov, Shaul Aloni, Tevye R. Kuykendall

**Affiliations:** 1 School of Science and Technology, Nazarbayev University, Nur-Sultan 010000, Kazakhstan; 2 The Molecular Foundry, Lawrence Berkeley National Laboratory, Berkeley, CA 94720, USA

**Keywords:** carbon nanotube, atomic layer deposition, dielectric, TiO_2_, nucleation layer

## Abstract

As one of the highest mobility semiconductor materials, carbon nanotubes (CNTs) have been extensively studied for use in field effect transistors (FETs). To fabricate surround-gate FETs— which offer the best switching performance—deposition of conformal, weakly-interacting dielectric layers is necessary. This is challenging due to the chemically inert surface of CNTs and a lack of nucleation sites—especially for defect-free CNTs. As a result, a technique that enables integration of uniform high-k dielectrics, while preserving the CNT’s exceptional properties is required. In this work, we show a method that enables conformal atomic layer deposition (ALD) of high-k dielectrics on defect-free CNTs. By depositing a thin Ti metal film, followed by oxidation to TiO_2_ under ambient conditions, a nucleation layer is formed for subsequent ALD deposition of Al_2_O_3_. The technique is easy to implement and is VLSI-compatible. We show that the ALD coatings are uniform, continuous and conformal, and Raman spectroscopy reveals that the technique does not induce defects in the CNT. The resulting bilayer TiO_2_/Al_2_O_3_ thin-film shows an improved dielectric constant of 21.7 and an equivalent oxide thickness of 2.7 nm. The electrical properties of back-gated and top-gated devices fabricated using this method are presented.

## 1. Introduction

Atomic layer deposition (ALD) is a technique predominantly used for the deposition of high-quality dielectrics. The ALD process occurs via a self-limiting reaction where the substrate is sequentially exposed to alternating pulses of two gas-phase precursors. This technique enables the deposition of thin films with excellent step coverage, angstrom-level precision and robust mechanical properties [1], making it an ideal choice for conformal coating of suspended and high aspect ratio structures. However, growth of the first few ALD layers on suspended single-walled CNTs is challenging due to the hydrophobic and inert nature of the nanotube’s surface. Usually, ALD deposition on suspended low-defect single-walled CNTs results in the formation of nanospheres, originating on nanotube surface defects [2,3]. The same problem exists for graphene [4,5] and 2D transition metal dichalcogenides [6,7], such as MoS_2_, WS_2_ and others. These materials do not have dangling bonds or surface groups on their basal planes and therefore no nucleation sites are available for the reaction of ALD precursors.

Currently, several surface functionalization techniques exist to promote ALD thin film growth on the surface of single-walled CNTs. Tubes can be chemically treated [8] with oxidizing agents, acids, bases, or annealed in plasma [9], which results in a chemical bond formation between functional groups and CNTs. Such covalent functionalization strategies generally create defects and additional scattering sites, as well as changing the carbon hybridization from sp2 to sp3. As a result of these surface modifications, the nanotube’s exceptional electronic and optoelectronic properties may degrade [10,11]. Another approach is to non-covalently attach different materials, such as DNA [12,13], surfactants [14], and polymers [15,16,17]. They do not distort the CNT lattice and do not influence the carbon hybridization. However, adsorbed molecules can change the local potential at the tube surface, creating scattering centers and inducing doping effects. These phenomena also influence CNT electronic and optoelectronic properties and may alter—but not necessarily degrade—device performance. For instance, NO_2_ functionalization can be used to prepare nanotube [18] or graphene [19,20] surfaces for ALD deposition of Al_2_O_3_; one of the first CNT gate-all-around transistors [21] was fabricated using this strategy. However, this method requires a NO_2_ source inside the ALD reactor, otherwise the physisorbed NO_2_ layer will desorb during sample transfer. It was also shown that the NO_2_ layer can strongly degrade CNT and graphene mobility due to the charged impurity scattering of the functionalization layer [19,21], which can be improved to some extent by annealing [22]. Although the above-mentioned non-covalent approaches solve the nucleation problem and promote ALD growth for electronics applications, the utilized materials should not reduce the overall dielectric permittivity of the gate oxide stack.

Another way to non-covalently prepare the surface of CNTs for subsequent ALD deposition is to first grow metal or metal-oxide seed layers. If a metal layer is used, it can later be oxidized in air at room temperature [23] or at elevated temperatures [24]. TEM measurements have shown that among commonly available metals, only Ti, Y, Ni and Pd show good wetting behavior on CNTs and result in continuous layers [25]. It was also shown that yttria, deposited by physical vapor deposition, can be used as both nucleation layer and high-k dielectric, delivering high transistor on/off ratio and an ideal subthreshold slope of 60 mV/dec [23,24]. The same strategy was used to prepare graphene [19,26,27,28] and MoS_2_ [29,30,31] surfaces. For graphene, it was also shown that TiO_2_ can be used as a buffer layer, improving the carrier mobility of the nanomaterial [28], whereas Al_2_O_3_ buffer layer may have an inverse effect [32]. It is also important to mention that non-covalent and covalent pretreatment strategies developed for graphene or other 2D materials cannot always be extended to CNTs, since the former represent planar structures with grain boundaries and edges—which makes both pretreatment and ALD deposition an easier task.

In this work, we present a method that enables the uniform and conformal coating of high-k dielectrics on defect-free single-walled CNTs by ALD. The method utilizes a Ti metal seed layer that is deposited by physical vapor deposition and oxidized to TiO_2_. The seed layer, attached to the defect-free CNTs enables subsequent conformal deposition by ALD. The ALD deposited Al_2_O_3_ dielectric coating on top of titania buffer layer, which is a high-k dielectric itself [33], results in a high-quality pure-oxide insulator. The study of the resulting thin film, as well as its influence on the CNT electronic and phonon properties are presented. The process is easy to implement, very large scale integration (VLSI) compatible, and results in deposition of high quality continuous ALD layers.

## 2. Results and Discussion

**Figure 1:**
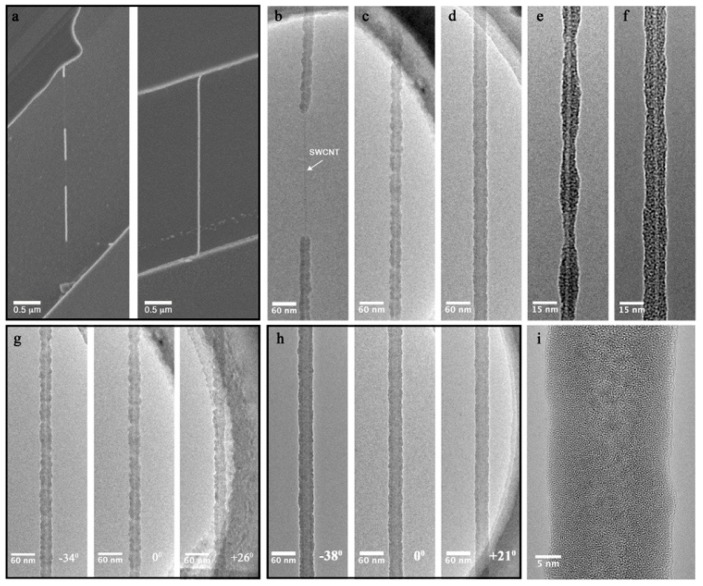
Morphology and coverage of CNT dielectric coatings. (**a**) SEM images of CNTs without TiO_2_ pretreatment (left) and with TiO_2_ pretreatment (right); (**b**–**i**) TEM images of CNTs with (**b**) 10 nm of Al_2_O_3_ only; (**c**) 3 nm of Ti converted to TiO_2_ and covered with 10 nm of Al_2_O_3_; (**d**) 5 nm of Ti converted to TiO_2_ and covered with 10 nm of Al_2_O_3_; (**e**) 3 nm Ti converted to TiO_2_ only; (**f**) 5 nm Ti converted to TiO_2_ only; (**g**) 3 nm of Ti converted to TiO_2_ with 10 nm of Al_2_O_3_, rotated over 60° along the nanotube axis; (**h**) 5 nm Ti converted to TiO_2_ with 10 nm of Al_2_O_3_, three different projections of the CNT rotated over 59° along the nanotube axis; (**i**) a corresponding high-resolution image of the CNT shown in (**h**).

To enable ALD deposition of Al_2_O_3_ on defect-free CNTs, a TiO_2_ nucleation layer was formed by deposition of Ti metal, which was oxidized to TiO_2_ under ambient conditions. To determine the effectiveness of the TiO_2_ pretreatment in promoting continuous, conformal coating by ALD, samples were prepared with and without the pretreatment and analyzed by SEM and TEM. Figure 1a shows SEM images of CNTs coated with 10 nm of Al_2_O_3_ by ALD without (left) and with (right) TiO_2_ pretreatment. Without the pretreatment, the CNTs were inconsistently and discontinuously coated, while with the TiO_2_ pretreatment, CNTs were consistently and continuously coated along their entire length. TEM analysis was carried out to further investigate the detailed structure of the coatings. Figure 1b shows a TEM image of a single-walled carbon nanotube (SWCNT) nominally coated with 10 nm Al_2_O_3_ by ALD without any surface pretreatment. As expected, the resulting thin film was discontinuous, with the deposited material most likely formed around rare surface defects or starting from the substrate and extended along the nanotube. When a titanium seed layer was deposited at a nominal thickness of 3 nm and oxidized, the TiO_2_ coating provided sufficient nucleation sites for subsequent ALD of 10 nm of Al_2_O_3_ (Figure 1c). The resulting coverage was continuous, but not uniform. To increase uniformity, a 5 nm Ti seed layer was deposited and oxidized. When coated with 10 nm of alumina by ALD, the coating was continuous, conformal, and the texture was smooth (Figure 1d).

To gain further insight into the morphology and uniformity of the pretreatment layer, further TEM analysis was carried out on CNTs coated with just Ti converted to TiO_2_. For the Ti layer, deposited by thermal evaporation in high vacuum, the mean free path (i.e., distance, which the evaporated material travels inside the chamber without colliding with gas molecules) is much larger than the metal target to substrate distance, making it a highly directional technique. This directionality results in the carbon nanotube shadowing itself and growth of thicker films on the part of the wall facing the evaporation front. As a result, a TiO_2_ layer with non-uniform thickness is formed and varies from about a nanometer on the shadowed side to a few nanometers. The sample with an initial 3 nm thick Ti layer (Figure 1e) resulted in a higher variation of film thickness in the longitudinal direction (pearling along the nanotube) when oxidized. However, slightly increasing the buffer layer thickness to 5 nm, resulted in higher TiO_2_ uniformity and continuous coverage of the nanotubes, although the resulting thin film still shows some thickness irregularities (Figure 1e). Figure 1g,h show the same Al_2_O_3_-coated nanotubes from Figure 1c,d, respectively—rotated ~60° along the nanotube’s longitudinal axis, which confirms that the CNTs are conformally coated. A high-magnification image of the CNT in Figure 1h is shown in Figure 1i, showing the uniform, amorphous ALD coating. 

Although the cause for the increased uniformity of the pretreatment layer from 3 to 5 nm is not completely understood, both nucleation layers showed complete coverage when coated with ALD deposited alumina. Continuous coating with TiO_2_ and later with Al_2_O_3_ can be explained as follows: due to a high binding energy, the initially deposited Ti layer has good wetting behavior on the nanotube surface [34], forming continuous (but not always uniform) layers. When oxidized, it grows significantly in size due to a high Pilling-Bedworth ratio (PBR_Ti_ = 1.6) [35]. As a result, enough nucleation sites for subsequent ALD reaction are provided to cover the entire nanotube. Some theoretical calculations show strong interaction between titanium and carbon, resulting in covalent bond formation between Ti and CNTs or graphene [36,37,38], which contradicts the idea of minimizing interaction between nanotubes and coatings. However, according to Density Functional Theory calculations, Ti is very reactive with O_2_ and oxidizes rapidly in its presence, significantly weakening Ti–C interaction upon oxidation [39,40]. Such theoretical considerations allow us to speculate that after oxidation, TiO_2_ only weakly interacts with the nanotubes. This is supported by Raman and the electrical measurements presented below, which do not show any degradation of the CNTs, although some changes are observed.

**Figure 2:**
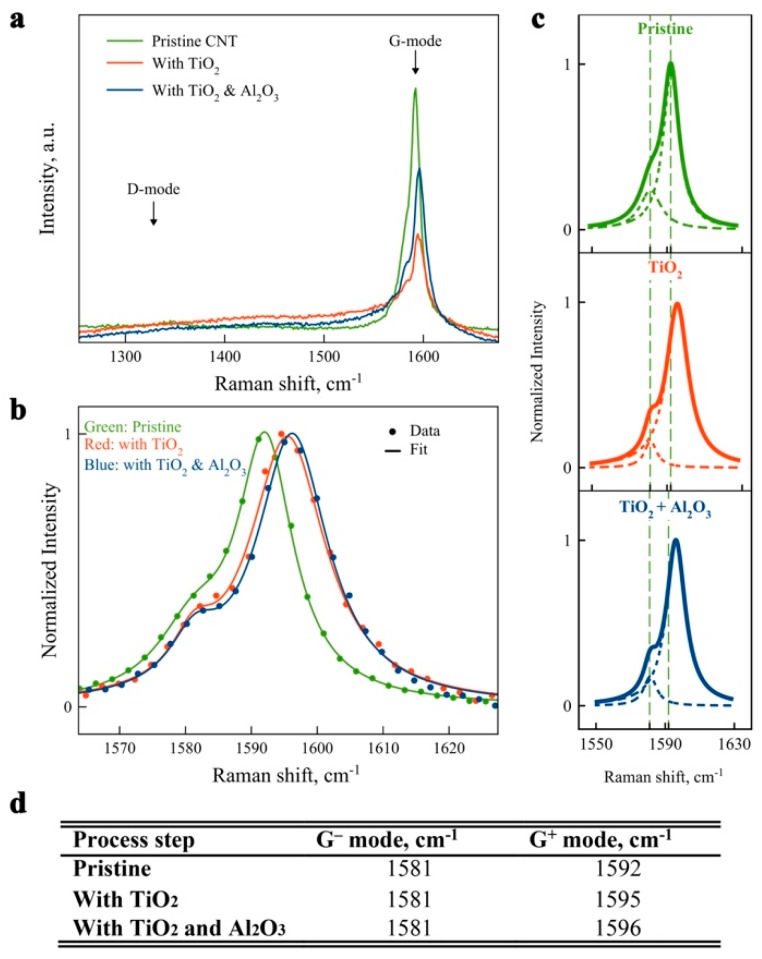
Effect of CNT coating analyzed by Raman spectroscopy before and after each deposition step: (**a**) as obtained G and D modes; and (**b**) G-mode region normalized and fitted with two Lorentzian line shapes for G**^−^** and G^+^ modes, separately shown in (**c**) for each spectrum; (**d**) A table showing G**^−^** and G^+^ modes’ peak positions and shift values (in parentheses) extracted from Lorentzian fits for each processing step. A Residual Sum of Squares of less than χ^2^ = 0.03 was obtained for all Lorentzian fits.

Raman spectroscopy was used to determine the effect of the coating on the phonon properties of the nanotube. Figure 2a shows Raman spectra obtained from the same nanotube after each step of the coating process: pristine CNT, after TiO_2_ deposition, and after Al_2_O_3_ coating. Raman spectroscopy shows no D-mode (or at the noise level) for all three experiments. This shows that initial nanotubes are low defect, as no defects were created during the pretreatment or ALD processes, and suggests that the technique does not degrade the quality of the CNTs. However, the G mode, responsible for carbon atom vibrations in circumferential (G^−^ mode) or parallel to nanotube (G^+^ mode) directions, underwent noticeable changes. Figure 2 shows that the peak of the G^+^ mode shifts toward the blue, relative to the pristine nanotube, for the TiO_2_ and TiO_2_/Al_2_O_3_ coated samples. The extent of the peak shift, determined by a Lorentzian fitting to the data, is shown in Figure 2c and summarized in the table in Figure 2d. This shift can be attributed to charge transfer and doping [41] or mechanical stress [42] induced as a result of TiO_2_ deposition. Subsequent coating with alumina showed a further small G-mode shift toward the blue. It is important to mention that such G peak position changes, with regard to pristine tubes, had a very large spread with both blue and red shifts across 20 tubes measured (results not shown here). We hypothesize that this can be attributed to mechanical stress induction, as nanotubes with different chirality show different Raman response at the same mechanical stress, demonstrating both blue (e.g., for uniaxial strain) and red (e.g., for torsional strain) shifts [43]. More research needs to be done to study deposition induced Raman modes shifts, which will help to understand the underlying phenomena and decouple different effects.

**Figure 3:**
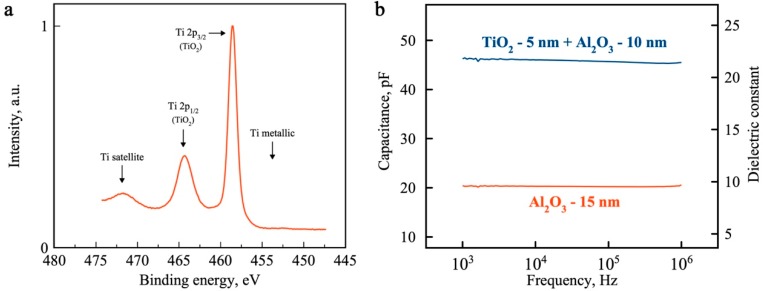
Chemical composition and dielectric properties. (**a**) XPS spectra of titanium layer oxidized in air for 24 h, confirming complete oxidation of the seed layer; (**b**) dielectric constant of pure ALD thin film (15 nm Al_2_O_3_, red curve) and compound dielectric (5 nm TiO_2_ + 10 nm Al_2_O_3_, blue curve) and their frequency response.

To verify that Ti is indeed converted to TiO_2_, X-ray photoemission spectroscopy (XPS) measurements were performed using a monochromatic Al Kα X-ray (hv = 1486.7 eV) source. To attain adequate XPS signal, larger samples were required and therefore carried out on a 5 nm Ti thin-film deposited on Si/SiO_2_, which was oxidized under the same ambient conditions as the CNT samples. Complete conversion to the oxide is important for transistor applications to suppress source-to-drain leakage currents via a metallic conduction pathway. In Figure 3a we see clear peaks at 458.5 and 464.3 eV, that correspond to Ti 2p_3/2_ (458.66 eV) and Ti 2p_1/2_ (464.31 eV), respectively [44], which have been identified as Ti^4+^ and correspond to stoichiometric TiO_2_. No peak was observed around 453.86 ± 0.32 eV, which would have been expected for metallic Ti [44], and is strong evidence that complete titanium oxidation was successful.

The dielectric quality of titania and alumina layers was also evaluated by fabricating thin-film metal-insulator-metal capacitors and performing capacitance-frequency measurements in the frequency range from 1 kHz to 1 MHz. Two capacitors with total oxide thickness of 15 nm were measured: first with 15 nm ALD Al_2_O_3_ and second with 5 nm TiO_2_ (oxidized Ti) plus 10 nm ALD Al_2_O_3_. Figure 3b shows the resulting capacitance-frequency characteristics. Despite having the same thickness and electrode area, the titania-alumina stack shows over two times higher capacitance (45.8 ± 0.3 pF) compared to the pure alumina capacitor (20.3 ± 0.1 pF). From a parallel-plate capacitor geometry, the alumina and average alumina-titania stack’s dielectric constants of 9.4 and 21.7 (at 1 MHz), respectively, can be extracted. Such an increase in k value can be explained by a high dielectric permittivity of TiO_2_. However, using only TiO_2_ as an insulator is not favorable, since the material has a relatively small band gap, which may result in thermionic emission and direct current tunneling [33]. Thus, for few-nanometers-thick dielectric layers, TiO_2_ should be used together with another high-k dielectric and a balance between these materials sought to optimize the overall thickness while maximizing the dielectric constant, and keeping leakage current low.

Another important metric in electronic device design, is the equivalent (silicon) oxide thickness (EOT). The EOT of our devices was calculated using the following equation: EOT = *ε_0_ε_SiO2_A/C_ox_*; where *ε_0_* is a vacuum permittivity, *ε_SiO2_* is a dielectric constant of SiO_2_, and *A* and *C_ox_* are the area and capacitance of the capacitor, respectively. For 15 nm thick films, an EOT of 6.2 nm was extracted for the pure-alumina device, whereas an EOT of 2.7 nm was extracted for the titania-alumina device (lower is better). Such a scale down of the EOT makes the proposed compound dielectric a promising candidate for future high-k dielectrics used in CNT- and other nanomaterials-based electronic devices. The quality of the interface between oxides, ratio of their thickness, as well as a combination of titania with other high-k dielectrics (e.g., HfO_2_, ZrO_2_) are subjects of future studies towards further improved EOT scaling.

**Figure 4:**
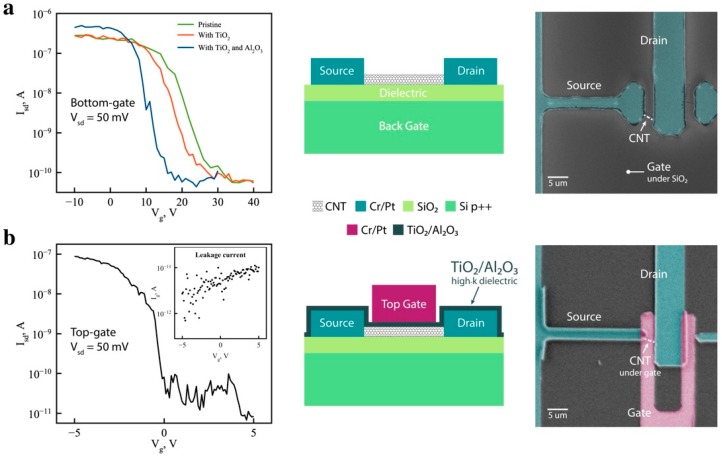
Electrical transport properties of CNT FETs. (**a**) Back-gated transport measurements of CNT devices, uncoated (pristine-green), with TiO_2_ only (red), and with TiO_2_ and Al_2_O_3_ (blue). A schematic illustration of the device geometry is shown (center), and a false-colored SEM image (right); (**b**) hole transport in top-gated CNT transistor. Insets in shows the leakage current. A schematic illustration of the device geometry is shown (center), and a false-colored SEM image (right).

Both back-gated and top-gated CNT field effect transistor (FET) device geometries were employed to probe the electrical transport properties of the CNTs. Back-gated devices on degenerate Si with SiO_2_ as the gate dielectric were employed to compare the TiO_2_/Al_2_O_3_ coated CNTs with uncoated-pristine CNTs, while the top-gated device allowed us to directly evaluate the TiO_2_/Al_2_O_3_ coatings as the gate dielectric. Back gated FET measurements confirmed that the TiO_2_ pretreatment technique does not degrade nanotube properties, nor does the subsequent Al_2_O_3_ coating. Figure 4a shows transport characteristics recorded from a pristine nanotube—a nanotube coated with TiO_2_—and a nanotube coated with TiO_2_ and Al_2_O_3_. The electrical measurement of the FET shows an on/off ratio of ~10^4^, with no degradation in conductance after TiO_2_ deposition and a slightly improved on/off ratio after the Al_2_O_3_ ALD deposition. The latter can be associated with annealing of the contacts during ALD processing at 300 °C. Device characteristics shift to negative gate voltages, becoming more p-type with each deposition step.

The possibility of using titania-alumina compound oxide as a high-k dielectric gate stack was evaluated by fabricating a top-gated CNT FET device. Figure 4b shows transport characteristics of the device. The observed differences in the threshold voltage between top and back-gated FETs is due to the difference in gate oxide thickness and dielectric constant: 15 nm thick TiO_2_/Al_2_O_3_ and 100 nm thick SiO_2_, respectively. As expected, both devices behave as p-type transistors due to the use of high work-function electrodes [45]. The electrical measurements of the top-gated device show an on/off ratio of ~10^4^ and the field effect mobility of the device was calculated using following equation: *µ_FE_ = g_m_ × L^2^/C × 1/V_ds_*, where *g_m_* is the transconductance, *L* and *C* are device length and capacitance respectively [46]. Capacitance per unit length was calculated as follows: *C/L = 2πε_0_ε_ox_r/2t_ox_*, where *ε_0_* is a vacuum permittivity, *ε_ox_* is a dielectric constant of the gate oxide (extracted from C-f measurements), *t_ox_* is its thickness, and *r* is the radius of the nanotube. The obtained field effect mobility of µ_FE_ = 226 cm^2^/Vs is comparable with those reported in literature, but far less than some of the “champions” in the field [47]. However, it is important to mention that field effect mobility is device-specific and many other effects have an impact on it, such as contact resistance, surface roughness, the quality of interfaces, measurement parameters, etc. The inset of I_sd_-V_g_ characteristics in Figure 4b shows low gate leakage current, which was limited by the sensitivity of measurement setup, and was observed at the noise level—at least an order of magnitude lower than the source-drain current. Such low leakage-current behavior, together with the extracted dielectric constant and EOT, show that the titania-alumina stack is a promising all-oxide dielectric. Further, in the top-gated FET, the bottom side of the tube was not covered with TiO_2_/Al_2_O_3_. In this configuration, the CNT was in contact with the hydrophilic SiO_2_ substrate, and water molecules on the surface likely interact with the nanotube, adversely impacting the CNT transistor performance [48]. Therefore, the fabricated transistor does not achieve its full potential benefit from the TiO_2_/Al_2_O_3_ high-k dielectric, and can be further improved by fabricating a surround-gate structure

## 3. Methods

### 3.1. Synthesis

The single-walled CNTs presented in this work were synthesized by chemical vapor deposition in a 1” quartz tube Lindberg/Blue M furnace reactor (Thermo Fisher Scientific, Waltham, MA, USA). A 2Å to 4Å thick Fe layer, was deposited on the substrate by thermal evaporation, then heated from room temperature to 675 °C in 30 min in air to calcinate the iron, followed by 2 min N_2_ purge to remove residual oxygen. After the purge, samples were further heated in 50 sccm flow of H_2_ for 20 min to reduce the iron and form catalyst nanoparticles. Once the CNT growth temperature of 875 °C was reached, CH_4_ at flow rate of 500 sccm was introduced. Nanotubes were synthesized for 30 min and then cooled to room temperature in H_2_ atmosphere. To verify the effectiveness of various coating methods and conditions, initial studies were carried on CNTs grown across ~3 μm trenches etched in Si/SiO_2_ wafers and analyzed by scanning electron microscopy (SEM). For TEM investigation, the nanotubes where grown over 1 or 1.5 µm circular holes directly on TEM support films. This process flow resulted in fabrication of single-walled carbon nanotubes, as evidenced by TEM.

Following the CNT growth, the suspended nanotubes were covered with Al_2_O_3_ both with and without titanium surface pretreatment. The titanium pretreatment consisted of thermal evaporation of titanium metal at a deposition rate of 0.1 Å/s and a pressure of 5 × 10^−6^ mbar or better; the titanium was oxidized to TiO_2_ by exposing samples to air for 24 h at room temperature. Native oxide is known to grow on titanium surface quickly—even at low temperatures [49]. Alumina layers were deposited using trimethylaluminum (TMA) and water precursors (Sigma Aldrich, St. Louis, MO, USA) at 300 ˚C in Oxford FlexAl ALD system (Oxford Instruments, Oxfordshire, UK).

To study the impact of the TiO_2_ layer or a TiO_2_/Al_2_O_3_ stack on the electronic properties of the CNTs, field effect transistors with bottom and top gates were fabricated on degenerately doped Si (p-type, R < 0.001 Ohm*cm) with 100 nm thermal oxide (UniversityWafer Inc, Boston, MA, USA). Standard UV photolithography was used to pattern device structures. Cr markers were deposited by thermal evaporation as alignment markers. Fe catalyst was deposited using thermal evaporation to define CNT growth areas. Nanotubes were grown using the synthesis methods discussed above. Lithography was again used to define source, drain and gate electrodes. Cr (2 nm) and Pt (60 nm) were deposited using an electron-beam evaporator. For the top-gated device TiO_2_/Al_2_O_3_ was deposited, followed by Cr/Pt gate electrode.

Dielectric properties of the TiO_2_ and TiO_2_/Al_2_O_3_ were evaluated by fabricating metal-insulator-metal capacitor stacks. This was done by synthesizing oxides, as described above, on a degenerately doped prime Si wafer that served as bottom electrode, and fabricating Cr/Au top electrodes using standard lithography and lift-off process. 

### 3.2. Characterization

To verify the continuity of the dielectric coatings over relatively long tube lengths, SEM was performed using a Zeiss Ultra 60 SEM (Carl Zeiss AG, Oberkochen, Germany) at an accelerating voltage of 2 kV. To study the morphology of the titania coating and the interface with the nanotube, TEM measurements were performed using a JEOL 2100F TEM (JEOL Ltd., Tokyo, Japan) at an accelerating voltage of 120 or 200 kV. The quality of the CNTs after each fabrication step was verified by Raman spectroscopy on Horiba Jobin Yvon LabRAM ARAMIS (Horiba Ltd., Kyoto, Japan) confocal microscope, with 532 nm Nd:YAG laser, focused by a 100× objective of 0.9 numerical aperture. X-ray photoemission spectroscopy (XPS) measurements were performed on a Thermo Scientific K-Alpha X-ray Photoelectron Spectrometer System (Thermo Fisher Scientific, Waltham, MA, USA), using a monochromatic Al Kα X-ray (hv = 1486.7 eV). The spectrum was obtained by integrating the Ti2p region 10 times, with a spot size of 400 um. A flood gun was used for charge compensation. The C1s carbon peak was used as an internal reference to compensate for charging effects. Peaks were fit using Avantage software (Thermo Scientific). The field effect transistor performance measurements were done using an Agilent 4156 Precision Semiconductor Parameter Analyzer (Agilent Technologies, Santa Clara, CA, USA) by recording the change in Source-Drain current while changing gate voltage at a constant source-drain voltage. A source-drain voltage of V_ds_ = 50 mV was used to avoid damaging the nanotubes during electrical measurements. Increasing V_ds_ to higher values resulted in nanotubes being burned in some devices. With a lower V_ds_, we were able to increase the probability that the devices survive the entire process, allowing electrical characterization before and after deposition of the dielectric. A Keysight E4990A Impedance analyzer (Keysight Technologies, Santa Rosa, CA, USA) was used to obtain capacitance-frequency characteristics and to study dielectric properties of the oxides in the frequency range from 1 kHz to 1 MHz at an amplitude of 0.01 V.

## 4. Conclusions

To summarize, we have demonstrated a method for achieving uniform ALD of high-k dielectric on low-defect suspended CNTs without degrading their properties. A few nanometer thick Ti layer, oxidized in ambient conditions to TiO_2_, was used to prepare the surface of inert single-walled CNTs for subsequent ALD coating of Al_2_O_3_. TEM measurements confirmed that the coatings were continuous and conformal, and Raman spectroscopy was used to show that the technique does not induce defects. We show that for thin-film structures, the TiO_2_/Al_2_O_3_ stack has a higher gate oxide dielectric constant relative to Al_2_O_3_ alone and exhibits a low EOT. FET devices were fabricated and showed the TiO_2_/Al_2_O_3_ dielectric stack to be an effective insulating layer with a low leakage current, and that the coatings do not degrade the properties of the CNTs. The process uses standard synthetic tools and is VLSI compatible. We believe that this conformal coating methodology will prove to be effective in the fabrication of surround-gate CNT FETs. This method may also find applications in a variety of difficult to coat nanoscale materials and devices, and the exploration of other ALD oxide materials could lead to dielectrics with further improved properties.
